# A cross-sectional study on health and physical functioning in relation to coping strategies among community-dwelling, ethnically diverse older women

**DOI:** 10.1186/1472-6874-10-10

**Published:** 2010-03-30

**Authors:** Luciana Laganà, Keren Zarankin

**Affiliations:** 1 Department of Psychology, California State University Northridge, 18111 Nordhoff Street, Northridge, California, USA

## Abstract

**Background:**

Although empirical evidence is available on the coping-health link in older age, research on this topic is needed with non-clinical samples of ethnically diverse older women. To contribute to filling such a research gap, we tested whether these women's general health and functional limitations were associated with specific coping strategies (selected for their particular relevance to health issues) and with known health-related demographics, i.e., age, ethnicity, income, and married status.

**Methods:**

In this cross-sectional study, respondents were recruited at community facilities including stores and senior centers. The sample consisted of 180 community-dwelling women (age 52-98) screened for dementia; 64% of them reported having an ethnic minority status. The assessment battery contained the Mini-Cog, a demographics list, the Brief COPE, and the Medical Outcome Study 36-Item Short-Form Health Survey.

**Results:**

Hierarchical multiple regression analyses showed that older women who used behavioral disengagement and, to a smaller degree, self-distraction as a form of coping reported lower levels of general health. The opposite was the case for positive reframing and, to a lesser degree, substance use. Moreover, lower income was related to worse general health and (together with more advanced age) physical functioning. None of the coping strategies achieved significance in the physical functioning model.

**Conclusions:**

These cross-sectional findings need corroboration by longitudinal research prior to developing related clinical interventions. Based on the initial evidence provided herein, clinicians working with this population should consider establishing the therapeutic goal of increasing the use of positive reframing while diminishing behavioral disengagement.

## Background

Health declines and functional limitations are common stressors for older women [1]. Chronic medical conditions, plus the risk of cancer, as well as heart and lung disease, all increase as women grow older, often exceeding the numbers presented by older men [2]. Additionally, compared to their male counterpart, older women typically have lower socio-economic status (SES), more inadequate assistance with basic life activities, and higher rates of medical care-seeking behaviors [3-5]. Under these hard-to-change circumstances, dealing with one's emotional responses to stressors, i.e., engaging in emotion-focused coping (as defined in the next paragraph), could be a reasonable choice. Indeed, many older women might not have the means to implement more active strategies when facing health challenges that often require health insurance and adequate income. Given the cultural-political context of the U.S., where ethnic minority groups are often in lower social positions, lack of health insurance and related health impairments are a common occurrence among older women from ethnic minority backgrounds [6]. Interestingly, some investigators have pointed out that coping strategies are even more important than income in their influence on health outcomes [7]. Thus, accounting for key demographics, geriatric researchers should investigate whether health outcomes in older age are linked to coping strategies potentially amenable to change via clinical interventions. This is a neglected research subject with regard to non-clinical samples of ethnically diverse older women. Testing whether available empirical evidence generalizes to this understudied population is needed, because most of the pertinent research findings are based on younger or medically ill samples. To minimize redundancies, we used the words "worse physical functioning" and "functional/physical limitations" interchangeably.

Concerning the conceptual framework of this study and the characterization of one of its key variables, Lazarus and Folkman's classic definition of coping is a cognitive and behavioral effort made by people for the purpose of reducing, minimizing, mastering, or tolerating the demands that rise from the interaction between them and their environment [8]. The authors postulated that coping has two major functions: dealing with the problem that is causing distress (problem-focused coping) and regulating emotions stemming from this problem (emotion-focused coping). In their theorization of the link between coping and somatic health status, Lazarus and Folkman envisioned three pathways through which physical health could be adversely affected by coping. Coping may: a) influence neurochemical responses and their characteristics (a topic beyond the scope of the present study), b) involve the excessive use of alcohol, drugs, or tobacco, which could have negative effects on health (thereby making substance use a possible health correlate), and c) regarding certain types of coping (such as avoidant mechanisms), impair health due to lack of adaptive health-related behaviors. In view of the last point, self-distraction (i.e., engagement in work or other activities to avoid thinking about a stressor) and behavioral disengagement (i.e., a reduction in a person's effort to deal with a stressor [9]) are potentially related to physical health.

We turned to prior literature to identify additional possible correlates of health outcomes in our target population, but found no definite consensus on the coping strategies best suited for this task. Research findings are mixed on the types of coping strategies related to better physical health; this is possibly due, at least partially, to the different ways (positive and negative) in which scholars define emotion-focused coping (a controversial topic beyond the scope of this study). In a meta-analysis on the coping-health link, Penley et al. found emotion-focused coping to be significantly related to negative overall health outcomes, with the opposite being the case for problem-focused coping [10]. Conversely, McGuinn observed that more recent research efforts show the salutary effects of emotion-focused coping strategies [11]. In particular, as people grow older, the use of such coping strategies seems to become more prevalent and potentially more beneficial [8,12,13], one more reason not to assume that findings of non-geriatric research in this area are generalizable to older women. Some scholars believe that emotion-focused coping is most frequently used in situations in which stressors are perceived as unchangeable [14] or as something to be endured [15]. This could very well apply to the aforementioned predicament in which many ethnically diverse older women find themselves in regard to their health and physical functioning, making the study of emotion-focused coping in this population a critical research target.

A cursory review of some of the relevant literature on the relationship between emotion-focused coping and health indicates that humor, often used to reappraise a stressful situation [16], had good potential for being related to older women's health outcomes, based on findings on other populations. Indeed, researchers have related humor to better physical health among both cancer patients [17] and older adults residing in assisted living facilities [16]. However, to our knowledge, there are no prior studies on this topic regarding community-dwelling, ethnically diverse older women. Positive reframing was also likely to be related to better health in our sample, considering Morse's findings that using this coping strategy in a health-promoting intervention for young women led to fewer reports of negative perimenstrual symptoms [18]. Some geriatric researchers have targeted positive reframing, but mostly within the context of spirituality/religiosity, a topic beyond the focus of our study. For instance, Gall discovered that various forms of religious coping involve the use of positive reframing, defined by the author as an active-cognitive coping strategy often leading to acceptance of illness [19]. We were unable to find empirical evidence specifically linking positive reframing to facets of health among community-dwelling older women. Regarding emotion-focused coping strategies that are potentially less adaptive, there is research evidence that self-distraction is a significant correlate of worse health outcomes and lower quality of life in heart failure patients [20], and that behavioral disengagement is a predictor of greater physical distress among cancer survivors [21], in line with Lazarus and Folkman's conceptualization [8] adopted herein. Moreover, findings on younger patients with health conditions such as psoriasis indicate that both behavioral disengagement and alcohol use are significantly and positively related to subjective physical disability [22]; once again, these findings might not generalize to our target population.

As for demographic factors typically correlated with health outcomes and thus needing to be accounted for when studying the coping-health link, advanced age is a well-known predictor of worse health [2]. Functional disability also increases with age (more in women than in men [23]), with more advanced age predicting declined physical performance [24]. Similarly, income is a known health correlate, as lower SES contributes to the development of a variety of life-threatening illnesses [25,26]. Additionally, researchers have found that unmarried status is related to worse health in non-European-American [27] and medically ill populations [11], and that having an ethnic minority background is a correlate of women's worse physical health [28].

Overall, the literature on emotion-focused coping strategies that are significantly related to health outcomes in non-clinical samples of ethnically diverse older women is limited. In particular, little or no research exists on how emotion-focused coping strategies and well-known demographic correlates of health contribute simultaneously to variance in this populations' physical health dimensions. As a step towards filling this gap in the literature, in the current study we chose to investigate emotion-focused coping strategies that have yet to be tested on such a population. Out of the available theoretical frameworks linking coping with health on which to base our selection of target variables, we chose Lazarus and Folkman's conceptualization of the coping-somatic health status link [8] (extended to physical functioning). Our choice of variables for this research was also based on prior empirical evidence on this topic, mostly from the study of other populations. Given such theoretical and empirical foundations, we hypothesized that five emotion-focused coping strategies and four known demographic correlates of health would be significantly associated with general health and physical functioning. Specifically, we tested the following hypotheses: 1) Humor and positive reframing would be related to better health and physical functioning; 2) Behavioral disengagement, self-distraction, and substance use would be associated with worse health and physical functioning; 3) More advanced age would be related to worse health and physical functioning; and 4) Higher income, European-American background, and married status would be associated with better health and physical functioning.

## Methods

### Participants

A total of 180 women (52 to 98 years of age) participated in this cross-sectional investigation, which was approved by the Institutional Review Board of California State University Northridge as part of a larger federally-funded study on older women's quality of life. The research participants were community-dwelling women recruited at places such as senior centers and stores; they all resided in Los Angeles County, California. The characteristics of the sample are illustrated in Table 1. Over 50% of the respondents did not attend college, and 64% of the sample self-identified as having an ethnic minority status. In Table 1, we tallied the ethnic categories as they were reported by each research participant, in order to best reflect respondents' self-identification with specific ethnic groups. Concerning participants' economic status, 33% of them lived below poverty level. The Institute of the Future [29] reported similar statistics concerning older adults living at or below poverty level in California (37%). Moreover, about 46% of the sample had a high school education at best, nearly mirroring the general population statistic for Los Angeles County [30] (47.3%). Based on the above statistics, our sample is likely to be representative of its population of origin.

**Table 1:** Characteristics of the sample

*Variable*	*Mean(Standard Deviation)*	*%*
Ethnicity		
European-American		36.1
Hispanic-American		27.8
Mixed ethnicity		13.9
Asian-American		8.9
African-American		8.3
Armenian-American		2.8
American Indian/Native American		2.2
Education		
Less than High School		29.4
Graduated from High School		16.7
Completed Trade School		5.6
Some college		23.9
Bachelor's degree		14.4
Some graduate school		1.7
Master's degree		6.7
Ph.D., M.D., and/or J.D.		1.7
Yearly Income		
Less than $20,000		33.3
$20,000-$39,000		34.4
Over $40,000		32.2
Marital Status		
Single		8.5
Divorced		16.1
Married		43.3
Widowed		32.2
Self-distraction	4.37(1.61)	
Substance use	2.24(.87)	
Humor	3.57(1.85)	
Behavioral disengagement	2.95(1.29)	
Positive reframing	5.02(1.82)	
General health	60.84(22.41)	
Physical functioning	62.26(27.12)	

Participants were required to be: over the age of 50, fluent in English, able to provide informed consent, and living independently. By excluding individuals residing in assisted living facilities, we minimized the possibility of recruiting older women with significant cognitive impairment, as further ascertained by using a dementia screener.

### Procedures

The procedures employed in this study were in accordance with the ethical standards of the aforementioned Institutional Review Board regarding research conducted on human subjects. Interviewers/research assistants recruited participants through purposive sampling (i.e., utilizing their connections within their ethnic communities) and snowball sampling (i.e., asking respondents to refer other women to this study). The focus of such recruitment strategy was to gather a sample as ethnically diverse as possible, including a combination of socially isolated women and those who had community contacts. This was done to increase the chances of the sample being representative of older women living in Los Angeles County, a very ethnically diverse area. The first author trained the interviewers in active listening, cultural competence, and interviewing skills. One-on-one assessment occurred at participants' homes or at locations close to their places of residence, such as libraries and senior centers. Each respondent received a code number that was placed on her assessment packet; names were not recorded to ensure confidentiality. The screening tool was then administered in order to rule out dementia. Over two assessment sessions, separated by a break to minimize fatigue, eligible women completed the assessment battery described below. The research assistants read the content of the measures out loud to each participant, to keep the assessment protocol as homogeneous as possible.

### Measures

Four measures were included in the assessment battery, i.e., the Mini-Cog, a demographic list, the Brief COPE, and the Medical Outcome Study 36-item Short Form Health Survey. First, potential participants were screened for dementia by administering the *Mini-Cog *[31], a tool that has been validated on a sample of ethnically diverse older adults [32]. This measure is comprised of an uncued 3-item recall test and a clock drawing test. Total scores of 3 or above suggest the absence of major cognitive disorders. We used this instrument exclusively for screening purposes and did not include its scores in the data analyses. All the women originally recruited received a total score of 3 or higher. Participants also completed a brief *Demographics List*, created by the first author, which contains 10 items that quantify variables such as age, gender, ethnicity, marital status, education, and income.

The *Brief COPE *[33] is a 28-item self-report measure used to assess specific coping strategies. Choosing this instrument was particularly appropriate, given that its development was based on the Lazarus and Folkman's conceptualization [8] adopted herein (as well as on a behavioral self-regulation model [34]). Respondents rated the frequency with which they had used each coping strategy for the purpose of managing stressful events within the last three months. The items in the Brief COPE are designed to measure 14 conceptually different coping reactions: active coping, planning, positive reframing, acceptance, humor, religion, use of emotional support, use of instrumental support, self-distraction, denial, venting, substance use, behavioral disengagement, and self blame. Each coping scale consists of two items with ratings provided on a 4-point Likert-type scale. The Brief COPE has demonstrated sound psychometric properties as a measure of both dispositional and situational coping efforts [9,33,35]. Moreover, it has shown to be highly reliable when used on multiethnic samples of women (e.g., obtaining a Cronbach's α of .86 in a study on ethnically diverse women living with breast cancer [36]). As already mentioned, to reasonably restrict the scope of this investigation, we targeted only a selection of coping strategies. This is methodologically appropriate, as Carver [33] stated that researchers can select coping scales of particular interest and this does not compromise the validity of the measure. The scales utilized in this study were self-distraction (Cronbach's α = .71), substance use (α = .90), behavioral disengagement (α = .65), positive reframing (α = .64), and humor (α = .73[33]).

We used the *Medical Outcome Study 36-Item Short-Form Health Survey *(MOS SF-36 [37]) to assess health outcomes. This instrument is suitable for use with an older population [38] and has good internal consistency (with a Cronbach's α >.80 for all MOS SF-36 scales [39]). It contains 36 items (with answers provided on a Likert-type scale) grouped into the following eight scales: physical functioning, bodily pain, role limitations due to physical health problems, role limitations due to emotional problems, general health, vitality, social functioning, and mental health. These eight scales can be categorized into two distinct domains, namely physical health and mental health, with physical health being comprised of general health, physical functioning, role limitations due to physical health problems, and bodily pain. The last two scales did not fit the scope of our study, thus we did not include them. Concerning the general health scale, respondents rated their current health and the extent to which they agreed with statements including their self-perception of vulnerability to disease. To assess physical functioning, participants were asked questions on whether their health limited them in carrying out a variety of vigorous, moderate, and light activities of daily living. In following the instructions provided in the MOS SF-36 manual [40], we used the MOS SF-36 software, which converted the raw data into standardized normed scores, then into *T *scores (with *M *= 50 and *SD *= 10). Ware et al. pointed out that not implementing this normative standardization step could negatively affect the reliability and validity of the MOS SF-36 scales. Table 1 contains the normalized transformed means and the standard deviations of this study's two health outcomes.

### Statistical Analyses

All the data analyses were carried out using the Statistical Package for the Social Sciences, version 12.0 (SPSS Inc., Chicago, IL). The descriptive statistics illustrated in Table 1 include percentages or mean values, depending on the nature of each variable, as well as standard deviations whenever applicable. Due to rounding, some of the total percentages did not add up to exactly 100. We also conducted zero-order correlational analyses (i.e., Pearson's) in order to quantify the bivariate relationships of all the independent and dependent variables. Moreover, two hierarchical multiple regression analyses were used to test our hypotheses within two regression models, one for general health and one for physical functioning. Each of the multiple regressions contained the same two blocks of variables, one for demographics and one for coping strategies. This procedure allowed us to test whether selected coping strategies accounted for a significant amount of variance in the total scores on each of the two health outcomes, while taking into consideration the contribution of demographics. Concerning the sample size required to properly conduct the regression analyses, Tabachnick and Fidell recommended that the minimum number of research participants needed for adequate power should be equal to 50 plus 8 times the number of independent variables [41]. Given that we had nine hypothesized correlates of general health and physical functioning, this number corresponded to 122 respondents in the current study (whose sample size exceeded it). Ethnicity and marital status were dummy-coded for use in the regression analyses (as recommended by Cohen et al. [42]), with 1 = European-American; 0 = Ethnic minority background; and 1 = married status; 0 = unmarried status, respectively. All research participants had complete data.

## Results

### Correlations among variables

Table 2 displays our findings on the intercorrelations of this study's variables. There was a strong relationship between the two outcome variables (which quantify two dimensions of health) and a moderate yet significant relationship between some of their hypothesized correlates, as discussed later. Moreover, age and behavioral disengagement were significantly and negatively associated with general health, indicating that respondents who were older and those who used behavioral disengagement reported lower levels of general health. Positive reframing was significantly and positively related to this health dimension, with participants who used this coping strategy reporting better general health. Age and physical functioning were significantly and negatively related, indicating that women of more advanced age reported lower levels of physical functioning. Conversely, income, married status, and positive reframing were significantly and positively associated with physical functioning, with respondents who had higher SES, were married, or used positive reframing reporting higher levels of physical functioning. Ethnic minority background was unrelated to health or coping variables.

**Table 2:** Zero-order correlations between the independent and dependent variables

*Variable*	Age	$	**Ethn**.	Married	**Self-distr**.	Sub.use	Humor	**Behav. diseng**.	**Posit. refram**.	General health	Physical **funct**.
Age	-										
$	.19*	-									
Ethn.	.11	.18*	-								
Married	-.21**	.29**	-.14	-							
Self-distr.	.03	-.06	.06	.02	-						
Sub. use	-.03	-.09	-.04	-.07	.06	-					
Humor	-.13	-.03	.04	.06	.38**	.18*	-				
Behav. diseng.	.03	-.01	-.05	.01	.17*	.20*	.17*	-			
Posit. refram.	-.19**	-.03	.07	.15*	.30**	-.11	.42**	.02	-		
Generalhealth	-.19**	.15	.08	.07	-.08	.05	.09	-.28**	.31**	-	
Physical funct.	-.33*	.21**	-.03	.17*	-.01	-.06	.03	-.11	.16*	.57**	-

*p < .05

**p < .01

### Multiple regression findings on general health

We then conducted a hierarchical multiple regression analysis to test the general health model. As shown in Table 3, more advanced age was significantly associated with lower levels of general health in Model 1. However, when we entered the demographics in Model 2 simultaneously with the five coping strategies, age was no longer significant, but income was. In the final model, positive reframing correlated positively with general health; the same was true, to a smaller degree, for substance use. Behavioral disengagement and, to a lesser extent, self-distraction were significantly related to lower levels of general health. The overall regression model accounted for a significant amount of variance in general health scores (Adjusted *R*^2 ^= .21). Because of the large number of independent variables, the Adjusted *R*^2 ^is reported herein.

**Table 3:** Summary of the hierarchical regression analysis for the general health model

	Model 1			Model 2		
***Variable***	***B***	***SE B***	***β***	***B***	***SE B***	***β***
Age	-.48	.20	-.18*	-.22	.19	-.08
Ethnic status	4.22	3.52	.09	1.77	3.23	.04
Income	2.48	2.20	.09	4.02	2.02	.15*
Married status	.93	3.56	.02	-1.00	3.27	-.02
Self-distraction				-2.05	1.03	-.15*
Substance use				4.23	1.82	.16*
Humor				.10	.96	.01
Behav. diseng.				-5.01	1.21	-.29**
Posit. refram.				4.47	.96	.36**
						
Adjusted *R*^2^		.04*			.21**	
*F *value		2.67*			6.37**	

*p < .05

**p < .001

### Multiple regression findings on physical functioning

The results of the second hierarchical multiple regression analysis (illustrated in Table 4) revealed that being older was significantly related to lower levels of physical functioning in Model 1. Once demographics were entered in Model 2 together with the coping strategies, age remained significant, and income became significant as well. In this analysis, none of the coping variables was significantly associated with physical functioning. The overall regression model accounted for a significant portion of variance in scores on physical functioning (Adjusted *R*^2 ^= .12).

**Table 4:** Summary of the hierarchical regression analysis for the physical functioning model

	Model 1			Model 2		
***Variable***	***B***	***SE B***	***β***	***B***	***SE B***	***β***
Age	-.94	.23	-.29**	-.87	.24	-.27**
Ethnic status	-.57	4.08	-.01	-1.73	4.14	-.03
Income	4.71	2.55	.14	5.19	2.59	.15*
Married status	3.50	4.12	.06	2.47	4.19	.04
Self-distraction				-.02	1.31	-.00
Substance use				-.47	2.33	-.01
Humor				-.42	1.23	-.03
Behav. diseng.				-2.08	1.54	-.10
Posit. refram.				1.79	1.24	.12
						
Adjusted *R*^2^			.12**		.12**	
*F *value			7.08**		3.66**	

*p < .05

**p < .001

## Discussion

In this cross-sectional study, we discovered that a variety of emotion-focused coping strategies were significantly related to general health. Among them was positive reframing, which was associated with better general health, as hypothesized. This result confirms Morse's findings on a sample of young women with health problems [18] and could reflect, at least partially, older women's dispositional optimism, which involves believing that good outcomes will occur in all kinds of situations, no matter how negative (such as having health problems). Dispositional optimism is a known correlate of better health among medically ill populations, as illustrated in a literature review on this topic [43]. Additionally, as predicted, behavioral disengagement and, to a smaller degree, self-distraction were associated with lower levels of general health. Such findings are in line with Lazarus and Folkman's conceptualization that avoidant mechanisms are likely to impair health because individuals who use them typically do not engage in adaptive health-related behaviors [8]. Furthermore, the behavioral disengagement result matches similar findings on cancer patients [21], and the one on self-distraction corroborates prior empirical evidence on heart failure patients [20].

Interestingly, substance use was significantly related to general health, but not in the expected direction, supporting previous research showing that older women drinkers report having better health than abstainers [44]. Perhaps the significant relationship of substance use with better general health is an artifact of cross-sectional designs, with older women in better health being able to indulge in substance use. The present finding does not necessarily conflict with Lazarus and Folkman's theoretical framework of the coping-health link [8], because in this conceptualization those authors referred to the deleterious effects of "excessive" substance use on health. It is possible that respondents who used this coping strategy consumed substances only in moderation, but we were unable to verify such a conjecture. Humor did not achieve significance, despite prior research showing its beneficial impact on health in institutionalized older adults [16]. As to demographics related to general health, more advanced age, a significant correlate of worse general health in Model 1, lost significance to income in the final model. These somewhat intertwined demographic findings could be due, at least in part, to the fact that age and income are typically interrelated in the geriatric literature, with levels of SES-based health inequalities increasing as a cohort ages [45]. Because age and income were significantly related in the present study (*r *= .19, *p *< .05), it is difficult to separate their weight in the variance explained in general health scores.

The second regression analysis yielded findings on coping strategies that were very different from those of the first regression. Not even one of the five coping variables achieved significance; only advanced age and, to a smaller degree, lower income were significantly related to worse physical functioning. The age result supports previous findings that, as people age, they report increased physical limitations [23,24]. Similarly, the income result confirms prior empirical evidence showing that declines in physical performance are significantly associated with lower income in relatively high-functioning older men and women [46]. Overall, the findings of this analysis suggest that older women's functional limitations are significantly related to the passage of time and to having limited income. Conversely, stronger financial means could provide increased ability to offset declines in physical functioning, for instance, by purchasing costly medications or helpful supplements, or by undergoing extensive physical therapy or medical treatment procedures that are effective at minimizing functional limitations. It should also be noted that ethnic minority background was unrelated to either coping or health variables. To offer one of the possible explanations (not empirically verifiable in the present study) of such a finding, perhaps this result was a function of all respondents being somewhat "Americanized". As participants were required to be fluent in English, they might have been acculturated to a similar degree, although it should be kept in mind that language fluency is only one index of acculturation, which was not directly assessed herein.

The current study has several limitations that could be addressed in follow-up research on health outcomes within our target population. Among them, due to the cross-sectional nature of this investigation, our results do not imply causation; longitudinal studies are needed to ascertain causality. The present findings might only be generalizable to the U.S. and be due to factors not assessed herein, including problem-focused coping strategies, acculturation, personality traits, having health insurance, formal medical diagnoses, and medication use, to name a few. Moreover, we did not assess how our research participants coped specifically with the stress of experiencing low levels of general health and physical functioning, mirroring the procedure adopted in prior coping research on medically ill populations [47]. We also did not address the fact that some bivariate correlations were significant; for instance, positive reframing was related to physical functioning (*r *= .16, *p *< .05), yet once this coping strategy was entered into the second regression model with the demographic variables, this was no longer the case. The potential reasons for such findings could be the focus of additional studies. Furthermore, we relied exclusively on participants' self-report, as done in the vast majority of the previously cited studies; additional research in this area could include more objective physical health information, such as data from medical charts.

## Conclusions

Age and income were significantly interrelated and were found to be strong correlates of general health and physical functioning among community-dwelling older women. Our results regarding emotion-focused coping strategies that are related to general health could be adequately explained using Lazarus and Folkman's conceptualization of the potentially deleterious effects of certain coping strategies on somatic health status [8]. In the present study, this conceptualization did not extend well to functional limitations. Also, not all prior research evidence on general health and physical functioning as they relate to the coping strategies tested herein was generalizable to our target population. The fact that the two regression analyses yielded such different results on coping supports the viewpoint held by some researchers that health should be measured multi-dimensionally [48]. Undoubtedly, the role of emotion-focused coping in manifestations of general health and physical functioning necessitates further demonstration. Nonetheless, our findings offer initial evidence that could be applied to clinical practice, as coping strategies are potentially modifiable variables. Fostering the use of specific coping strategies for the enhancement of well-being might be incorporated as a therapeutic goal when providing clinical care to this study's target population. Among others, a cognitive-behavioral psychotherapeutic approach could be adopted, identifying (in addition to other relevant clinical issues) key situations in which to apply, for instance, positive reframing of illness-related stressors [19] such as having to depend on others or experiencing discomfort during medical procedures. Additionally, more effective behavioral choices may be outlined and subsequently reinforced, to help patients be better prepared to deal with health-related stressors that, upon arising, might otherwise trigger the automatic utilization of coping strategies that could make the situation worse.

## Competing interests

The authors declare that they have no competing interests.

## Authors' contributions

LL: Principal investigator, devised this study, conducted all the data analyses, and wrote the paper. KZ: Provided assistance during data analyses and wrote a first draft of this paper. Both authors read and approved the final manuscript.

## Pre-publication history

The pre-publication history for this paper can be accessed here:

http://www.biomedcentral.com/1472-6874/10/10/prepub
